# Poverty, user fees and ability to pay for health care for children with suspected dengue in rural Cambodia

**DOI:** 10.1186/1475-9276-7-10

**Published:** 2008-04-25

**Authors:** Sokrin Khun, Lenore Manderson

**Affiliations:** 1 National Centre for Health Promotion, Ministry of Health, Phnom Penh, Cambodia; 2 School of Psychology, Psychiatry and Psychological Medicine, Faculty of Medicine, Nursing and Health Sciences, Monash University, Clayton, Australia

## Abstract

User fees were introduced in public health facilities in Cambodia in 1997 in order to inject funds into the health system to enhance the quality of services. Because of inadequate health insurance, a social safety net scheme was introduced to ensure that all people were able to attend the health facilities. However, continuing high rates of hospitalization and mortality from dengue fever among infants and children reflect the difficulties that women continue to face in finding sufficient cash in cases of medical emergency, resulting in delays in diagnosis and treatment. In this article, drawing on in-depth interviews conducted with mothers of children infected with dengue in eastern Cambodia, we illustrate the profound economic consequences for households when a child is ill. The direct costs for health care and medical services, and added indirect costs, deterred poor women from presenting with sick children. Those who eventually sought care often had to finance health spending through out-of-pocket payments and loans, or sold property, goods or labour to meet the costs. Costs were often catastrophic, exacerbating the extreme poverty of those least able to afford it.

## Background

Resource-poor countries have long struggled to control infectious disease, reduce mortality and severe morbidity, and improve childhood survival rates with inadequate resources that are echoed in delayed diagnosis and poor service delivery at local levels. From the 1980s, various financing systems have been introduced to supplement government budgetary allocations, loans and bilateral aid, to deter the unnecessary use of health services, and from 1988, to improve access to and quality of services. The Bamako Initiative in particular emphasised the introduction of user fees, specifically to supplement the budgets of local health facilities, meeting salary and supply shortfalls, and so improve access and quality.

In an review published a decade ago, still apposite, Gilson notes the lack of attention to the ability of poor households to pay fees, and the effects of user fees on health seeking and treatment [1]. Subsequent studies in low and middle-income countries on the relationship between user fees and the utilization of public health services support claims that direct costs discourage presentation by poor people [2-5]. In Ghana, user fees have been shown to discourage presentation for antenatal and midwifery care, and consequently contribute to continued high maternal and neonatal mortality [6]. In Tanzania, despite general willingness to pay when quality of care at lower-level health facilities was improved, the very poor, women and elderly were negatively affected [7]; in Niger, user fees also resulted in declining patients' attendance and variable cost recovery [8]. The current view therefore is that minimally, there need to be effective safety nets for poor and vulnerable households, with alternative approaches to user fees such as micro-health insurance and/or exemptions and waivers from cost sharing [7,9,10].

These studies, like those cited by Gilson [1,11], have largely been conducted in Africa. Yet despite the local impact of the Bamako Initiative (1988), health financing reforms to supplement government funds have been introduced far more extensively. One study in Laos produced similar results to the African studies, drawing attention to the negative impact of user fees on the very poor [12], but relatively little research has been conducted in Asia on user fees and health seeking behaviour. This is particularly important when early diagnosis and treatment is critical for individual outcomes and where prompt medical care is a cornerstone of control. This is the case for various infections, including acute respiratory infection, tuberculosis, malaria, and dengue fever, which predominate in resource-poor settings.

Appropriate funding mechanisms are necessary to ensure equitable access to health care. The costs of health care to consumers, and the sources from which money is derived to pay for health care, are therefore important from a policy perspective, because the health care system in Cambodia is heavily based on fees. In Cambodia, user fees were introduced in 1997 as a component of a broader suite of health care reforms introduced to generate cash to supplement the basic low salaries of health staff, so to motivate staff and discourage them from seeking unofficial payments from patients or undertaking extra jobs during official working time. Cash generated from user fees was also expected to contribute to the operational costs of health facilities, so improving the quality of health care. To ensure that the reforms would not prevent poor people from using government health facilities, the Ministry of Health gave local health workers discretionary power to provide partial or full fee-exemptions for health care to people deemed particularly vulnerable [13]. The impact of this on service use appears to be uncertain. In 2006, 18%, 16% and 9% of people received user-fee exemptions from health centres, provincial referral hospitals and national hospitals respectively [14]. Attendance at public hospitals appears to have increased, but this may be not only because official hospital fees are lower than those charged by local private health practices, but also because they are lower than the informal fees still charged by government health workers, including for drugs and other supplies not available at the facilities [15-18].

A particular risk for people living in poverty, for whom health expenditure can be catastrophic, is that health may be traded off against other immediate needs. Because of the vulnerability of very poor householders, in 2000, in conjunction with development partners, the Cambodian Government introduced two types of social safety nets: a health equity fund and a community-based health insurance scheme. Currently, the health equity scheme covers 35 of 73 operational districts nationwide, and provides funds to cover the user fees of people who are defined as poor by subjective assessment. The community-based health insurance scheme has been piloted in parts of the country by local non-government organizations. The average premium is around US$5 per family per annum, and is used to pay for public health care services at health centres and referral hospitals. There are no schemes that cover fees at private health care centres, although regardless of financial status, many Cambodians use these preferentially.

The Cambodia Demographic and Health Survey in 2005 [19] indicated that the direct costs of treatment were high. The calculations, including transportation, food, medication, and administrative, pathology and other fees, indicate that the average cost of a single illness episode was US$15.52 for public facilities, US$18.62 for private services, and US$6.25 for non-medical services (e.g. purchasing local drugs and/or visiting shamans, fortune tellers, Buddhist monks and traditional healers). There were small disparities in the sources of money spent on health care by provider. Although coverage through the health equity fund or community-based health insurance helped some people, still there were substantial demands on people related to health seeking from both the public and private sectors and including non-medical expenses. These cash demands were covered variously by wages or earnings from casual and informal activities (42% to 65%), and by savings (20% to 32%) and loans (14%).

Other studies support these findings of the extent of out-of-pocket expenses. In India and Vietnam, people similarly used cash reserves to meet most health care costs [20,21]. In Kampong Cham (KPC), eastern Cambodia, people spent an average of US$11.87, with 39.1% of the expenses met by wages and other cash resources, 39.6% from savings, 13.3% from formal loans and 7.8% from friends [19]. This is the area in which this study was conducted. This article draws on data collected as part of an ethnographic study of social aspects of dengue fever (DF), which remains one of the top 10 reasons for hospitalization in the country, and an important cause of child mortality. Prevention and control activities for the disease are conducted by the National Dengue Control Program (NDCP), under the management of the National Malaria Centre of the Ministry of Health. The NCDP is responsible for planning, implementing, monitoring and evaluating dengue control activities nationally, although at the Operational Health District and Health Centre (village) levels, dengue control is integrated into a "package" of communicable diseases, including malaria and schistosomiasis. We have described prevention and control activities elsewhere [22,23]; here, our focus is on the costs of care and the effect this has on the ability of villagers to respond to episodes of illness.

## Methods

The research on which this article draws was conducted over eleven months (March 2003 – February 2004) in two villages of KPC, which we refer to as Khun and Nekry and which had populations of 1195 and 615 people respectively. These villages were purposively selected because of the consistent high prevalence of DF, despite their inclusion as NDCP operational areas for Abate (temephos) distribution and health education campaigns, and despite the presence of village health volunteers and the inclusion of dengue prevention in the school curriculum. Each village had a public health centre, private health practitioners and traditional healers; each village also was located (in opposite directions) approximately 30 km from the provincial capital and referral hospital, accessible by buses and motor bike taxi (*motor dup*).

Data were collected by the first author using both qualitative and quantitative methods. In order to explore government activities to control DF, these methods included participation in a government workshop on dengue distribution in provinces which had a high prevalence of DF, document analysis, interviews with policy-makers, health administrators, and service providers at national, provincial and district levels, and the observation of health education, Abate distribution, and routine health clinics. Key informant interviews were conducted with village health volunteers and other community members about common health problems, health seeking behaviours, and the costs of medical treatment. Focus group discussions were conducted with mothers of children infected with dengue on health seeking behaviours and cost of treatment. In-depth interviews were also conducted with all but two women whose children had been infected in the past year or during the research period (N = 29) about their patterns of seeking treatment of dengue fever, medical treatment at both private and public health facilities, financial sources, ability to pay for health care, and their attitudes to user fees charged at government health facilities. These data were supplemented by village maps, field notes, the analysis of health educational material on DF produced by NGOs and the National Dengue Control Program, a school survey undertaken in the primary schools in both villages, and by entomological surveys to identify *Aedes *mosquito breeding sites, larval density and village practices regarding water containers and hard waste disposal [22-24].

In this article, we focus on data generated from interviews with women who had children who had been sick from DF (N = 28), with one woman who had lost three of her five children from dengue shock syndrome, and with managers and staff at village health centres, and provincial and national levels (N = 15). With verbal permission, interviews were tape-recorded. Field notes were written up as soon as possible after interviews or group discussions, and subsequently all recorded materials were transcribed and translated into English. Coding was undertaken with attention to chronology and diversity of actors and triggers to action in the narrative analysis of individual accounts, and to identify shared themes, commonalities and diversity of opinion across interviews and in relation to other data sources. SPSS version 11.0 was used to manage and analyse quantitative data from interviews and household surveys, the entomological surveys, school survey and observational checklists. Ethics approval was provided by the Human Research Ethics Committee of The University of Melbourne, the Ethics Committee of WHO/TDR, and the National Ethics Committee of the Ministry of Health, Cambodia. In this article, we attribute statements by context (interview = Int; focus group discussion = FGD) and have assigned a number to each participant.

## Results

We begin with the one woman who had lost three of her five children to fever, possibly dengue. She was vague about the cause of death in all cases because, she said, she "knew nothing," but she was clear about the role of poverty in their deaths:

*At the beginning, she (her first daughter) had a mild fever. I didn't know what I was; I didn't know that it was dengue (*krun chiem*). I bought *para* (Paracetamol) for her, then I felt her – she was cold and it was already the second or third day and she couldn't talk. So then I took her to the health centre and told them about her condition and that she was always sleeping. They gave me enough tablets for a day. The next day she became worse and was always sleeping. I couldn't take her for a blood test and I didn't know what to do. I bought medicine locally and I kept her until she died. At the time, I had no money. I couldn't do anything although I thought of everything (to work out what to do). My daughter was dead ... this is because I had no money. When there is no money, people can't do anything... money is needed for everything.... The same thing happened with my second child, maybe due to dengue ... he seemed to have a mild fever but then the blood cells erupted and the doctor couldn't give him an injection, and he died too ... (the next one) was six months old and had cold extremities. I didn't know if it was dengue. But when she had fever, I ran for help everywhere in the village and they told me to take her to the doctor. But I didn't take her to the hospital. I didn't have the money to pay up front at the provincial hospital (Int 22)*

In 2004, when the field research was drawing to an end, an estimated 35% of the population of Cambodia (of c. 14 million) were living below the poverty line, defined as US$0.50 and US$0.45 per capita per day in urban and rural areas respectively [14]. Around 90% of this population lived in rural areas where income generation was precarious, because of changes in land tenure, increasing indebtedness and landlessness, lack of irrigation facilities, poor harvests, few alternative sources of employment, and limited access to markets. Those engaged in subsistence production often lacked cash for everyday expenses: school fees, transport, clothing, fuel for cooking and the purchase of foodstuffs not produced locally, as well as health care and medical provisions. As we illustrate below, the high costs of health care and medical services often exacerbated the poverty of those least able to afford it.

### Expenditure on health care for dengue fever

Our focus on dengue fever as an instance of health expenditure derives from its prevalence. In 2003, when the study commenced, DF, DHF (dengue hemorrhagic fever) and DSS (dengue shock syndrome) were among the top causes of morbidity and mortality in Cambodia. In the first eight months of 2007, as we revisited this data, Cambodia was experiencing the most dramatic outbreak of the disease in recent years: nationwide, there had been 34,542 cases and 365 deaths from DF, DHF and DSS; in KPC there were 5105 cases of the disease and 65 deaths [25]. Control of dengue, and reduced severe morbidity and mortality, depend on the early recognition of signs of disease, prompt presentation for diagnosis, and appropriate medical care. We have illustrated elsewhere that delays were often substantial [24]. As we describe below, these delays are at least partly due to the lack of available cash in the event of illness.

During the study period (2003–2004), the severity of dengue fever when the child was brought to a provider determined the length of hospitalization, treatment procedures, amount of and kinds of medicine, and therefore the total cost of care. Regardless of whether they used a public or private facility, villagers reported spending on average US$34.50 and up to US$150 for a single episode of dengue, with those not granted a fee exemption spending a mean of US$49.29 (range US$25–150) at a public facility and a mean expenditure of US$34.60 (range US$8.75–50.00) at a private practice. This amount related to direct and immediate indirect expenses including medical advice, medication, and travel and food for health care outside their villages, although not indirect costs such as loss of income. Only two of eight women who took their children to the referral hospital in KPC received exemptions from paying the user-fee. For example, one woman in Khun village spent approximately US$30 when her child was admitted to the public children's hospital in KPC for suspected DF in 2003; during three days of admission in the facility, she spent US$6 for the bed fee and serum, US$6 for drugs and syringes which were unavailable in the hospital, US$10 for ice to cool the child and for food for the child and other family members, and around US$8 for motorbike transportation to and from the town. Another woman who took her child to the provincial referral hospital spent less than others only because her child stayed in the hospital for a relatively short period and she received partial exemption, paying US$5 instead of the scheduled fee of US$10 to US$25 depending on services: *"I had no money to buy food; I spent it all on serum that I bought myself and the doctor only did the perfusions. I bought injections and syringes. I had no money for a bed fee. I had to tell my mother to stay and look after my child in the hospital and I came back to find (borrow) money" (Int 20).*

These costs are exceptionally high, and catastrophic in rural Cambodia. In Khun and Nekry villages in 2003–4, the *mean *income was estimated as exactly on the poverty line (of US$13.50 per month), and so significant numbers of people were living on less. But at the same time, the cost per illness episode could be exorbitant. Without fee-exemptions, at public facilities, villagers reported spending on average US$49.29 (range US$ 25–150) and at private practices, on average US$34.60 (US$ 8.75–50). These estimated costs include a consultation fee and medicine, and transport and food when health care was sought outside of the village (see Table 1).

**Table 1:** Expenditure and sources of money for treatment of children with DF

**Place of health facilities**	**Expenditure**	**Period of illness**	**Sources of money**
**Public facilities**			
Referral Hospital	US$25	1 episode	Savings, loan from mother
**Combined private and public facility**			
Local private practice, referral hospital	US$150	1 episode	Sale of rice
Local private practice, referral hospital	US$62.50	1 episode	Loan,* sold farm land
Local drugs, HC, referral hospital	US$37.50	1 episode	Pawn farm land for 3 years
Local drugs, referral hospital	US$37.50	1 episode	Not specified
Local private practice, referral hospital	US$31.25	1 episode	Loan
Local drugs, referral hospital	US$25	1 episode	Loan, sale of a pig fee-exemption
Local private practice, referral hospital	US$3.75	1 episode	Fee-exemption
**Private Practices**			
Local drugs, private practice, blood test	US$100	2 sons, 2 episodes	Sale of farm land
Local drugs, local private practice	US$50	1 episode	Sale and loan*
Local drugs, private practices in the village and in KPC	US$50	1 episode	Not specified
Local drugs, private practice	US$45	2 sons, 2 episodes	Sale of an ox
Local drugs and private practice in KPC	USS37.50	1 episode	Sale of a pig
Local drugs, private practice, blood test	US$37.50	1 episode	Not specified
Local drugs, private practice, blood test	US$36.25	1 episode	Loan from mother
Local drugs, local private practice	US$25	1 episode	Loan
Local drugs, local private practice	US$25	1 episode	Sale of an ox
Local drugs, local private practice	US$ 8.75	1 episode	Savings, loan from mother

* Loan for more than a year, the exchange rate was 4000 riels = USD 1 at the time of the study in 2003.

Most residents in the villages and surrounding areas were farmers, supplementing income from rice with the sale of other agricultural and horticultural products, the occasional sale of livestock, and the occasional resale of non-food goods purchased in larger quantities from KPC or Phnom Penh. As illustrated in Table 1, in both Khun and Nekry, the main source of money to meet health costs was loans and the sale of property, goods and labour. Of the 12 women who specified the source of money to meet medical and related costs, the majority (8/12) received loans from their neighbours or a local money lender, depositing property, farm land or cattle as security. Loans tended to be free of interest if under US$25, provided they were to be repaid within days. If the amount was greater and the repayment period extended, villagers paid interest at rates of 15–20% per month. Many villagers could not afford to repay the loan and interest, and simply continued to pay accruing interest at an annual rate of 180–240%. The high interest payments whittled away any cash that came to hand, and the continued depletion of material resources. Villagers could not repay both interest and capital without selling assets such as their farmland, cattle or house. One woman, working as a casual labourer for around 4000 riels (c.US$1) a day in the rainy season, and selling firewood for 500 riels ($0.13) a bundle, explained how she pawned her farmland for five *chi *(gold measurement, 1 chi = US$35 in 2003) to pay for the medical costs for her daughter, spending all her money on food, medical treatment and transportation. Another woman, who had taken her child to the provincial hospital, recalled:

The dengue fever was severe on the fourth day, and they wouldn't let me take him home. I had no money, so I deposited land for a loan to pay for food, medicine and other expenses. So now I have no land and I have to do other things to earn money to buy rice for my children. If I earn 2000 riels, I spend 2000 riels; if I earn 3000 riels, I spend 3000 riels (Int 5).

Health workers also reflected that "money was a problem during dengue outbreaks:"

The family took the child to hospital. The wife had to come home for money, and the husband could not make enough money from carting water to sell to other villagers, so it was very difficult for them. The farming was not fruitful, they did not want to go to the doctor, they tried to do whatever they could, and they hoped that it would get better. But it's just too difficult for the poor ... They committed suicide (FGD 30).

### User-fee exemptions

User fees are set, in theory, on the basis of consultations among HC staff and community members, with approval from senior staff of the district hospital and provincial health department. National policy specifies that one percent of the total revenue collected is to be remitted to the Ministry of Finance, 49% is to supplement the running costs of the health facility receiving the fees, and the remaining 50% is to supplement staff salaries as motivation. The rates are displayed on a board hanging at the entrance of each building, and vary from US$0.25 cents for a consultation to US$3.75 for a normal delivery. In both Khun and Nekry HCs, the revenue approximately US$60 was raised from fees each month, resulting in each staff member receiving a supplement of US$5 a month to their official monthly salary of US$10–15. In the referral hospital in the provincial capital, the fee ranged from US$2.5 for administration and consultation to US$50 for surgery. Staff members received approximately US$25 monthly from the fees supplement their monthly salary of US$20 – 35, although this still fell short of the amount needed to meet basic living expenses and was often less than the cost of a single visit to a hospital for medical advice and treatment. In the study villages, health workers estimated that they needed around US$150 per month for food, clothes and medical costs for a family of five; in KPC, around US$200 per month. Consequently, health workers continued to accepting informal payments from patients at the government clinic or took on additional jobs: opening a private health practice at home; farming; or operating a motor taxi (*rot motor dup*).

Although user fees only partially met the shortfall between salaries and living costs, they were important to health workers. While in theory exemptions were granted on the basis of poverty reported by fellow community members, exemptions were relatively arbitrary and tended to be granted by sympathetic health workers who knew of specific circumstances or in response to personal appearance indicating abject poverty. Students, monks, village health volunteers and village development committee members were also granted exemptions from the user-fee. In 2003, the annual exemption rate at HC of Nekry and Khun was 26.34% and 42.82% respectively, indicating that a significant number of people were receiving exemptions from fee payments at the village level, in contrast to the referral hospital in KPC, where the annual exemption rate was 16.71% [26] (and even this compared well with the exemption rate of the National Maternal and Child Health Care in Phnom Penh of 4–7%). An exemption constituted lost income for the health facility and for staff, and should have been covered by the central government or an NGO. However, in 2003 fees were not reimbursed either to the HCs or the referral hospital in KPC.

While exemptions in Khun and Nekry were comparatively high, many extremely poor villagers still had to pay user fees, and according to participants in focus group discussions and in-depth interviews, user fees were a major obstacle for the very poor to seek health care at a public facility. Care at the government HC required up front payment if they were not granted exemption, but many people had neither cash nor valuables to secure a loan. Poor villagers therefore had limited access to public health care because, as one government health official admitted, the user fees were rarely lifted for them and patients were discouraged from presenting because of the multiple costs incurred:

It's difficult for the poor. In the public hospital they need to pay a lot...a user fee, bed fee and food, and the child might still die... Money is a problem during dengue outbreaks ... I told a client to take the child to hospital. She said no, because one of her children had already died two days after admission. She said she'd rather let the child die at home than take it to hospital. Some people are very poor. When I refer them, they cry because they have no money and want to be treated at the health centre, but they can't because the health centre can't keep people for more than 12 – 20 hours and the patients need to be referred because I'm afraid of shock (FGD 30).

Several villagers considered the fees at public health facilities to be a financial barrier to seeking health care from public health centres in the villages. A mother explained:

One time I took my elder child to the HC. They gave me medicines, then I said I had no money. One of the staff members got very angry. I don't dare go there any more, unless I have money. I went there again when I had 500 riels. I saw the staff member who had been angry with me, and I couldn't enter the HC (Int 8).

Instead, villagers sought advice locally or in KPC from private practitioners who they could pay over time. Although any treatment involved money, private health practices provided women with the option to pay by instalment. While some private practitioners were medically trained, they were often qualified nurses or midwives who worked in local HCs and ran small practices from their own house to supplement their income: that is, they were the same people who the women would have seen had they presented at the government centre. The two local practices in Nekry and Khun were both located on the ground floor of private houses. The practice room was a simple set-up: a small cupboard with medicines and other basic supplies – paracetamol tablets, bags of serum, vitamins, antibiotics, antiseptic fluid, cotton bandages, cotton wool and strips – purchased from pharmacies in KPC or Phnom Penh, or distributed by Cambodian pharmaceutical companies. The costs of services were the same whether villagers presented to the private practitioner at home or requested a house call, and were fairly standard throughout the region; the consultation, with sufficient medication to last for 2–3 days, was approximately 2,000 riels. The most expensive services were for medical injections and serum perfusions, the latter commonly administered to residents who believed that it reduced fever and cured disease. Each injection cost 2,000 riels and each serum perfusion 40,000 riels a litre. Each perfused serum bag was mixed with injectable drugs, or more often, simply with glucose or vitamin C, allowing practitioners to charge additional money again.

In an emergency, as happened if dengue shock syndrome developed, women often took their child to the provincial government hospital in KPC:

*My son had high fever so I pressed *nonong*leaves to make juice for him, then I got *peth*(doctor) Ek Chun to give for injections. My son became worse with a higher fever, so my father in-law told me to take him to the *peth*in KPC. My husband told him that we had no money, so my father in-law gave us 10,000 riels to take my son for a blood test at the hospital in KPC. The staff told me that my son had typhoid fever and dengue. He was hospitalized for five days. When we were discharged, we gave the doctor 30,000 riels. He accepted only 5,000 riels and returned the rest to us. He asked us if we had money to return home. My husband said we had no money, so he gave us the money. I don't know why; perhaps he felt sorry for us because my husband is blind (Int 10).*

While this woman received partial exemption, other villagers faced very large bills. Villagers knew that services were cheapest at the village HC, and that in KPC, the cost was lower at public health centres than in mixed private and public services. But they also knew that if they presented to the referral hospital, they would have to pay upfront. In an emergency, they had no time to sell their belongings or get a loan, but they would often not have enough money to pay the hospital admission fee and so would have difficulty getting their child examined and admitted. Apart from the scheduled fees, most interviewees reported having to pay additional costs such bed or laboratory fees, and to buy medicines, catheters or blood if these were not available at the hospital. Transport, food, accommodation and other costs, and lost income, again influenced decision making. One woman noted:

You need a number at hospital to get to see a doctor. Sometimes we have to wait too long... one of my neighbours spent a few days (at the children's hospital) and came back empty-handed ... just waiting for the number, just waited and waited, and spent one week waiting – then got nothing. When I heard that – I have no money to wait (Int 26).

These additional costs increased the financial burden for patients and deterred people from utilizing this facility. Many people, like the woman who lost three of her five children, kept their children at home, using home remedies and waiting for the fever to resolve itself when village treatments failed.

## Discussion

All people who participated in this study – villagers whose children had had dengue fever, and their health providers, spoke of lack of money recurrently, always returning to this as they narrated their own and others' stories of illness, hospitalisation and sometimes death: "No money, no medicines (Int 23)," one woman explained; another, "Without money, I daren't go there (hospital) (Int 8); and another, "It's very difficult when we have no money" (Int 5).

The fee-exemption rates of 18% and 16% at public health centres and referral hospitals in Cambodia is relatively low compared to other developing countries, where exemption rates range from 20% to 93% [3,27]. In the study area, in contrast, they were comparatively high, but as discussed above, exemptions were given not only to the poor and not necessarily to the poor. Individuals (and their immediate families) who had work connections with the facility, and students and monks, were exempted, but numbers of people who were poor, on any objective basis, were compelled to pay fees. The health centres and hospitals had limited incentives to treat poor people free of charge because they would lose revenue from the fees and the reimbursement mechanisms were not operating. The introduction of user fees consequently led service providers to enforce fees wherever possible, rather than to ensure access to care as a priority.

As already discussed, given the current poverty status in Cambodia, people have to sell property or borrow to pay for health care. The categories of user fees set at the National Children's Hospital and National Maternal and Child Health Care were very similar to those operating in provincial and district levels, with quality of health care also varying substantively depending on ability to pay. People with more money could afford better health care in private air-conditioned rooms; those with less money received a different standard of health care in a shared ward; those without had to wait for a fee subsidy from a non-government organization or the sympathy of someone in a health facility. If a family could not afford the travel costs to take their ill child to the facility, then they had no option but to keep their child at home, test home remedies, and wait to see the outcome.

Due to different health seeking behaviours, the costs of health care met by respondents in Khun and Nekry were much higher than the average costs of US$11.87 spent by the population in KPC on health care, although less than those of villagers in Banteay Meanchey, northwest Cambodia, where medicine and medical treatment were far more expensive because of the locality of the province in a cross-border commercial zone. In this area, according to van Damme and colleagues [17], villagers who used private services first, then public ones, paid from US$6 to US$97 with an average of US$32, 81% of which was paid in the private sector and the rest at the public hospital.

Despite safety net mechanisms introduced to prevent inequity, in practice the service fees and the necessary purchase of diagnostic supplies, serum, drugs and other materials punish the poor. The policy itself aims to raise money from the poor to finance a health system that tends to overlook them. With the introduction of fees for public health care, villagers have had to pay more not less in direct costs, in addition to indirect costs such as transport and food, opportunity costs (lost wages), and fees charged by private practitioners in cases when they used these before resorting to the public facility. During this period of delay, while the child is infectious, others in the village are also at a risk of infection. In this respect the user fees result in the iatrogenic transmission of dengue fever and illustrate how "iatrogenic poverty" is induced by the health system [17,28].

## Conclusion

Cambodia suffers the highest infant, child and maternal mortality in Southeast Asia, and dengue fever continues to contribute to this. Dengue fever causes substantial out-of-pocket expenses and creates substantial socio-economic burdens for families in KPC and elsewhere in Cambodia. Data from this study indicate that mothers' perceptions of the cost of health care and the lack of financial resources result in significant delays in seeking health care [24]. The data also show that the introduction of the user-fee and limited application of fee exemptions severely impact on the utilization of public health facilities. Finally, the direct and indirect costs of health care aggravate poverty. The possibility of removing or changing the systems of application for user fees is a huge challenge for policy makers, given their needs also to improve the quality of health care at public health facilities. If the user-fee is maintained for income generation, public funds need to be available and accessible to cover all those who are genuinely poor.

## Competing interests

SK conducted this research while a PhD student in the School of Population Health, The University of Melbourne. He holds a permanent position with the National Centre for Health Promotion of the Ministry of Health, Cambodia, and was on leave from this job while undertaking the study on the community participation in the prevention and control of dengue in Cambodia.

## Authors' contributions

SK and LM jointly conceived of the project and contributed to its design. All fieldwork and data management was conducted by SK. Both authors made substantive contributions to this paper and approved the final manuscript.

## References

[B1] Gilson L (1997). The lessons of user fee experience in Africa. Health Policy Plan.

[B2] Ridde V (2003). Fees-for-services, cost recovery, and equity in a district of Burkina Faso operating the Bamako Initiative. Bulletin of the World Health Organization.

[B3] Nyonator F, Kutzin J (1999). Health for Some? The Effects of User-Fees in the Volta Region of Ghana. Health Policy Plan.

[B4] Collins D, Quick J, Musau S, Kraushaar D, Hussein I (1996). The fall and rise of cost sharing in Kenya: the impact of phased implementation. Health Policy Plan.

[B5] McIntyre D, Thiede M, Dahlgren G, Whitehead M (2006). What are the economic consequences for households of illness and of paying for health care in low- and middle-income country contexts?. Soc Sci Med.

[B6] Witter S, Arhinfu DK, Kusi A, Zakariah-Akoto S (2007). The experience of Ghana in implementing a user fee exemption policy to provide free delivery care. Reproductive Health Matters.

[B7] Bonu S, Rani M, Bishai D (2003). Using willingness to pay to investigate regressiveness of user fees in health facilities in Tanzania. Health Policy Plan.

[B8] Meuwissen LE (2002). Problems of cost recovery implementation in district health care: a case study from Niger. Health Policy Plan.

[B9] Abdu Z, Mohammed Z, Bashier I, Eriksson B (2004). The impact of user fee exemption on service utilization and treatment seeking behaviour: the case of malaria in Sudan. Int J Health Plann Manage.

[B10] Kivumbi GW, Kintu F (2002). Exemptions and waivers from cost sharing: ineffective safety nets in decentralized districts in Uganda. Health Policy Plan.

[B11] Gilson L, Mills A (1995). Health Sector Reforms in Sub-Saharan Africa - Lessons of the Last 10 Years. Health Policy.

[B12] Paphassarang C, Philavong K, Boupha B, Blas E (2002). Equity, privatization and cost recovery in urban health care: The case of Lao PDR. Health Policy Plan.

[B13] Cambodia Department of Planning and Health Information (1998). Guidelines for Developing Operational Districts.

[B14] World Bank (1993). World Development Report: Investing in Health.

[B15] Jacobs B, Roberts E (2004). Baseline assessment for addressing acute malnutrition by public-health staff in Cambodia. J Health Popul Nutr.

[B16] Akashi H, Yamada T, Huot E, Kanal K, Sugimoto T (2004). User fees at a public hospital in Cambodia: effects on hospital performance and provider attitudes. Soc Sci Med.

[B17] Van Damme W, Van Leemput L, Por I, Hardeman W, Meessen B (2004). Out-of-pocket health expenditure and debt in poor households: evidence from Cambodia. Trop Med Int Health.

[B18] Soeters R, Griffiths F (2003). Improving government health services through contract management: a case from Cambodia. Health Policy and Planning.

[B19] Cambodia Ministry of Planning, Cambodia Ministry of Health (2005). Cambodia Demographic Health Survey, 2000.

[B20] Harving ML, Ronsholt FF (2007). The economic impact of dengue hemorrhagic fever on family level in Southern Vietnam. Danish Medical Bulletin.

[B21] Roy K, Howard DH (2007). Equity in out-of-pocket payments for hospital care: Evidence from India. Health Policy.

[B22] Khun S, Manderson L (2007). Abate distribution and dengue control in rural Cambodia. Acta Tropica.

[B23] Khun S, Manderson L (2007). Community and school-based health education for dengue control in rural Cambodia. PLoS Neglected Tropical Diseases.

[B24] Khun S, Manderson L (2007). Health seeking and access to care for children with suspected dengue in Cambodia: An ethnographic study. BMC Public Health.

[B25] Cambodia NDCP (2007). Dengue Surveillance Statistics in Cambodia, 2007.

[B26] Cambodia Kampong Cham Referral Hospital (2004). Annual Health Report of the Kampongcham Referral Hospital in 2003 (in Khmer).

[B27] Bank W, UNFEM, ADB, UNDP, DFID/UK (2004). A Fair Share for Women. Cambodia Gender Assessment.

[B28] Meessen B, Van Damme W, Tashobya CK, Tibouti A (2006). Poverty and user fees for public health care in low-income countries: lessons from Uganda and Cambodia. Lancet.

